# Thalamic Functional Connectivity during Spatial Long-Term Memory and the Role of Sex

**DOI:** 10.3390/brainsci10120898

**Published:** 2020-11-24

**Authors:** Dylan S. Spets, Scott D. Slotnick

**Affiliations:** Department of Psychology and Neuroscience, Boston College, Chestnut Hill, MA 02467, USA; sd.slotnick@bc.edu

**Keywords:** anterior thalamus, mediodorsal thalamus, sex differences, gender differences, functional connectivity, gPPI

## Abstract

The thalamus has been implicated in many cognitive processes, including long-term memory. More specifically, the anterior (AT) and mediodorsal (MD) thalamic nuclei have been associated with long-term memory. Despite extensive mapping of the anatomical connections between these nuclei and other brain regions, little is known regarding their functional connectivity during long-term memory. The current study sought to determine which brain regions are functionally connected to AT and MD during spatial long-term memory and whether sex differences exist in the patterns of connectivity. During encoding, abstract shapes were presented to the left and right of fixation. During retrieval, shapes were presented at fixation, and participants made an “old-left” or “old-right” judgment. Activations functionally connected to AT and MD existed in regions with known anatomical connections to each nucleus as well as in a broader network of long-term memory regions. Sex differences were identified in a subset of these regions. A targeted region-of-interest analysis identified anti-correlated activity between MD and the hippocampus that was specific to females, which is consistent with findings in rodents. The current results suggest that AT and MD play key roles during spatial long-term memory and suggest that these functions may be sex specific.

## 1. Introduction

Two subregions of the human thalamus most often implicated in long-term memory are the anterior thalamic nucleus (AT) and mediodorsal nucleus (MD) [1]. AT receives direct projections from the hippocampus along with other medial temporal lobe structures including the fornix and the mammillary bodies and is considered part of the “extended hippocampal system” [1,2]. Abnormalities in AT have been identified in the prodromal phase of Alzheimer’s disease and have led to evidence suggesting that amnesia presented in Alzheimer’s patients is due to neurodegeneration of the Papez circuit (which includes AT, subregions of the medial temporal lobe, and the posterior cingulate cortex) [3], which underlines the importance of AT in episodic memory [4]. Although AT is predominantly associated with the hippocampus (c.f. [1,3]), connections between AT and the prefrontal cortex via the anterior thalamic radiation have more recently been identified through the use of diffusion tensor imaging [5]. In one study, deep brain stimulation to AT produced a significant improvement in performance during a verbal recall test [6]. In a separate study, unilateral exocytotic lesions to AT (with ipsilateral ablation of the inferiotemporal cortex/hippocampus in the opposite hemisphere) produced deficits in learning tasks that required integration of objects and spatial locations in the contralesional hemifield [7]. These results suggest AT is important in many forms of memory, including spatial memory.

Early speculation about the involvement of MD in long-term memory stems from Korsakoff patients [8], who have lesions to this thalamic nucleus and present with amnesia. However, as is often the case, lesions in many of these patients were not restricted to MD and often involved other thalamic nuclei, including AT [9]. Based on the potential involvement of MD in long-term memory, anatomic connections to and from this nucleus have been mapped in rodents and non-human primates. These connections include dense reciprocal connections between MD and the prefrontal cortex, including the lateral, dorsal, and medial prefrontal cortices [10]. Lesions to MD in non-human primates have been linked to deficits in spatial working memory [11] and object-in-place discrimination [12]. Case studies of unilateral MD lesions in humans have linked this nucleus to both visual and verbal long-term memory in both recall and item recognition tasks [13]. 

Functional connectivity analyses in humans have identified increased connectivity between MD with subregions of the medial temporal lobe. In one study, participants indicated their degree of familiarity (on a scale from 1 to 3) with previously studied faces, objects, or scenes [14]. In addition to these familiarity ratings, participants also indicated whether they “recollected” the relevant stimulus or whether it was “new”. Activity in MD was associated with familiarity strength across all three types of stimuli, suggesting that MD plays a role in material-general familiarity. Activity in AT, however, was consistently associated with recollection (versus strong familiarity) across all three types of stimuli, suggesting that AT plays a role in material-general recollection. In a subsequent functional connectivity analysis, MD was found to be functionally connected (i.e., a functionally connected activation produced by a connectivity analysis) with the perirhinal cortex and the parahippocampal cortex. Moreover, the degree of connectivity with these regions was found to vary with the strength of familiarity (with greater connectivity between these regions indicating a greater sense of subjective familiarity). In contrast, AT was not found to be functionally connected with any subregions of the medial temporal lobe. In another study, subjects studied face–scene pairs and, at retrieval, indicated which of three faces was originally paired with the scene of interest [15]. Contrary to the results of Kafkas et al. [14], Geier et al. [15] did not find any difference in the strength of functional connectivity between MD or AT with subregions of the medial temporal lobe as a function of memory accuracy; however, MD did have greater connectivity to the hippocampus, perirhinal cortex, and parahippocampal cortex than AT.

AT and MD have been theorized to support parallel processes during declarative memory, where AT is thought to support the selection of memory contents and MD is thought to support the selection of retrieval strategy [16]. The roles that each nucleus plays in human memory, however, are still widely debated, with some suggesting that MD and AT work in parallel to support memory retrieval [10] and others suggesting that each nucleus plays a separate role (such as familiarity versus recognition as discussed above; see [14,15,17]).

Mounting evidence suggests that sex differences exist during long-term memory. Functional magnetic resonance imaging (fMRI) studies have reported sex differences in the patterns of brain activity across a variety of long-term memory types including object recognition [18,19,20,21], facial recognition [22,23], autobiographical memory [24,25], and spatial memory [26] (for a review see [27]). A recent meta-analysis of sex differences in long-term memory studies identified greater activity in the lateral prefrontal cortex, visual cortex, parahippocampal cortex, and the cerebellum for males compared to females [27]. Greater activity for males than females has also been observed in the hippocampus during spatial memory [26], autobiographical memory [24], and virtual maze navigation [28]. One study that investigated the relationship between hippocampal lateralization and retrieval strategy during long-term memory found that greater activity in the left hippocampus was associated with a verbal retrieval strategy in females, whereas greater activity in the right hippocampus was associated with a visual retrieval strategy in males [19]. Thus, females appear to be more likely to utilize a verbal strategy during long-term memory retrieval, whereas males are more likely to utilize a visual–spatial strategy during long-term memory and are more likely to engage the hippocampus (cf., [29]).

Preliminary evidence from rodents suggests that the thalamus may modulate sex differences in hippocampal activity during long-term memory. Specifically, inactivation of the thalamic–hippocampal pathway rescued hippocampal activity and memory performance in female mice, but not in male mice [30]. This suggests that the thalamus may inhibit the hippocampus during long-term memory in females, which may explain the comparatively greater hippocampal fMRI activity for males described above.

Although the functional connectivity of the human thalamus has been investigated during a resting-state task [31] and during non-spatial long-term memory [14,15], to our knowledge, the functional connectivity of the thalamus during spatial long-term memory has not been investigated. The aims of the current investigation were twofold: (1) to identify functional connectivity with AT and MD during spatial long-term memory and (2) to identify whether sex differences exist in the patterns of whole-brain connectivity with each nucleus. We expected AT and MD to produce a network of connections that included regions with known anatomic connections with each nucleus as well as regions that support long-term memory such as the prefrontal cortex, parietal cortex, visual processing regions, hippocampus, and parahippocampal cortex [32]. Moreover, based on the findings in mice that suggest inhibition of the hippocampus by the thalamus in females [30], we hypothesized that the magnitude of activity in the hippocampus and thalamus during spatial long-term memory would be anti-correlated in females (but not in males).

During encoding, abstract shapes were presented to the left and right of fixation. During retrieval, shapes were presented at fixation, and participants made an “old-left” or “old-right” judgment. We identified spatial memory hit-versus-miss activity in AT and MD and conducted a functional connectivity analysis, using activations in these two nuclei to determine which brain regions were functionally connected to the thalamus. To preview the results, activations functionally connected to AT and MD existed in regions with known anatomical connections to each nucleus as well as in a broader network of long-term memory regions, and sex differences were identified in a subset of these regions.

## 2. Materials and Methods

The present study reanalyzed two spatial long-term memory studies, each comprised of two experiments [33,34]. Essential methodological details are provided here (for full details, see [26]).

### 2.1. Participants

There were 40 female and 18 male participants across the two studies. Eighteen females were selected from the 40 female participants to best match the spatial memory accuracy and variance of the 18 male participants. Eleven of the 18 females and males were drawn from Study 1 [33] and the remaining females and males were drawn from Study 2 [34]. Critically, participants were matched on spatial memory accuracy and variance within each experiment such that female and male performance were matched within each experiment, and an equal number of females and males were drawn from each experiment.

### 2.2. Stimulus Protocol and Task

**Figure 1:**
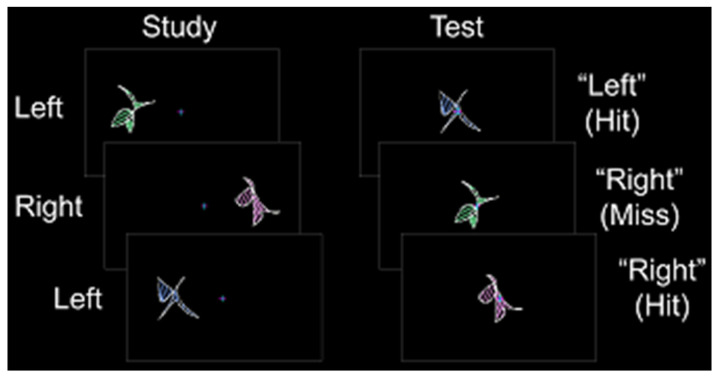
Stimulus and response protocol. During the study phase, abstract shapes were presented to the left or right of fixation. During the test phase, old shapes were presented at fixation, and participants indicated whether each shape was previously on the “left” or the “right”.

Prior to the scanning session, each participant completed a behavioral training session. Participants also completed a single anatomic scan and a variable number of study/test runs. In Experiments 1 and 2, participants completed three study/test runs. In Experiments 3 and 4, participants completed either seven or eight study/test runs. During each study phase, abstract shapes were presented in pseudorandomized order to the left or right of a fixation cross for 2.5 s (shape construction details can be found in [33]). Participants were instructed to remember each shape and its spatial location while maintaining fixation. Each shape was presented at fixation for 2.5–3.0 s during the test phase (a constant duration for each experiment), and participants made an “old-left” or “old-right” judgment followed by confidence judgment with their left hand (Figure 1). Spatial memory accuracy was calculated as the percentage of correct spatial location identification contingent on correct old-item identification (chance = 50%). Latin square counterbalancing was used to assign shape sets across participants.

### 2.3. Image Acquisition and Analysis

In Experiments 1 and 2, images were acquired using a 3-Tesla Siemens Allegra MRI scanner (Siemens, Erlangen, Germany) with a standard head coil. Anatomic data were acquired using a multiplanar rapidly acquired gradient echo (MPRAGE) sequence (TR = 30 m, TE = 3.3 m, 128 slices, 1 × 1 × 1.33 mm resolution). Functional data were acquired using a T2*-weighted echo-planar imaging sequence (TR = 2000 m, TE = 3.3 m, 64 × 64 acquisition matrix, 26–30 slices in Experiment 1 and 30 slices in Experiment 2, 4.5 mm isotropic resolution). In Experiments 3 and 4, image acquisition parameters were identical to those in Experiments 1 and 2 except that a 3-Tesla Trio scanner (Siemens, Erlangen, Germany) was used with a 32-channel head coil and that 33 slices and a 4 mm isotropic resolution were used to acquire the functional data. 

A random-effect general linear model (GLM) analysis was conducted in SPM12 (Wellcome Trust Center for Neuroimaging, London, UK; https://www.fil.ion.ucl.ac.uk/spm/software/spm12/). Functional image preprocessing included slice-time correction, motion correction to the first volume of each run, and spatial normalization to the Montreal Neurological Institute (MNI) template, which included resampling at 2 mm^3^. Anatomic images were normalized to MNI space with 1 mm^3^ resolution and then averaged across participants. The following event types were entered into the GLM: encoding of items in the left visual field, encoding of items in the right visual field, accurate retrieval of items in the left visual field (left-hits), accurate retrieval of items in the right visual field (right-hits), inaccurate retrieval of items in the left visual field (left-misses), and inaccurate retrieval of items in the right visual field (right-misses). To maximize power, events were collapsed across confidence responses. For each participant, activity associated with accurate spatial memory was isolated by contrasting spatial memory hits (i.e., left-hits and right-hits) and spatial memory misses (i.e., left-misses and right-misses).

AT and MD were identified as two regions of interest (ROIs) based on our a priori hypotheses regarding their functional connectivity with the whole brain as well as with the hippocampus during memory retrieval. Notably, the use of anatomically-defined (or in this case, guided) ROIs is permissible in functional connectivity studies given strong hypotheses regarding specific regions. To avoid selecting noisy voxels in the anatomic ROIs, task-related activity within these ROIs guided the selection of voxels that were used as seed regions for the subsequent connectivity analysis. Each participant’s first-level model was entered into a second-level random-effect GLM analysis. The contrast of hits versus misses (inclusive of all participants) was first thresholded at *p* < 0.01 (without cluster extent correction) to identify whether there were any activations in AT or MD, which were to be used as a seed for the psychophysiological interaction analysis. There was one activation within the MD ROI at this threshold (see Section 3, Results). A more lenient threshold of *p* < 0.05 was then applied to identify activity within the AT ROI, and there was one activation in this region at this threshold (see Section 3, Results). The most significant voxel of activity within each of these regions was used to define the center of a 3 mm radius sphere for the generalized psychophysiological interaction (gPPI) analysis. Locations of AT and MD seeds were confirmed using a statistical probabilistic atlas map created using a large sample of participants from the Human Connectome Project (for full methodological details on the construction of the atlas, see [35,36]).

Functional connectivity analyses were conducted using the gPPI toolbox ([37]; https://www.nitrc.org/projects/gppi) using the individual participant first-level models of hits versus misses. For each participant, whole-brain t-contrasts of AT and MD functional connectivity were created and entered into two separate second-level models, across participants, to determine voxels functionally connected to AT and MD. Second-level models were evaluated with independent t-test to determine whether there were any sex differences in the magnitude of connectivity. The gPPI toolbox first extracts time courses of activity from a specified seed region (in this case each AT and MD ROI). This creates a vector for each time point in the dataset for a particular seed region. This seed region vector acts as a regressor in a subsequent GLM analysis. Voxels in other brain regions that have a significant temporal correlation with the seed region are identified as regions that are functionally connected to the seed region [38].

All functional connectivity contrasts were thresholded at *p* < 0.01, cluster extent corrected to *p* < 0.05. To compute the cluster extent threshold, we first computed the spatial autocorrelation for the gPPI contrast of hits versus misses for all participants in each experiment and employed the smallest spatial autocorrelation value (3 mm) across experiments. We conducted 10,000 Monte Carlo simulations based on the acquisition volume parameters, spatial autocorrelation, and the desired individual voxel and family *p*-value [39]. This resulted in a cluster extent of 24 voxels. This cluster extent was applied to all functional connectivity contrasts. Although it has been claimed that cluster extent threshold correction for multiple comparisons can have a relatively high false-positive rate [40], this method of correction has been shown to produce acceptable false-positive rates (see [39,41] for a critical evaluation of [40]). Images were imported into MRIcroGL (nitrc.org), overlaid on the average anatomic for viewing purposes, and an exclusive white matter mask was employed.

In addition to the standard GLM analysis described above, a targeted/hypothesis-based ROI analysis was conducted to determine whether there was a negative relationship between the magnitude of hippocampal and thalamic activity. First, to identify whether there were any connected activations (i.e., a functionally connected activation produced by the gPPI analysis) in the hippocampus for females, we conducted the gPPI contrast of hits versus misses for MD and AT with only females. The MD contrast produced one connected activation in the hippocampus for females that was negative in magnitude (*p* < 0.01, cluster extent corrected to *p* < 0.05; see Section 3, Results). A 3 mm sphere was extracted around the peak of this hippocampal activation, which was used as the hippocampal ROI for the correlation analysis. For both ROIs (in MD and the hippocampus), beta weights were extracted for each participant using custom scripts written in MATLAB (MathWorks, Natick, MA, USA). To test for sex differences, the same procedure was repeated for males, using the hippocampal ROI identified for females. For each participant of a given sex, the average beta-weight value across spatial misses was subtracted from the average beta-weight value across spatial hits. This created a spatial hit − miss beta-weight value for each ROI for each participant. The spatial hit − miss beta-weight values in the hippocampus were plotted as a function of spatial hit − miss beta-weight values in MD for each of the sexes, and a Pearson correlation was conducted. A one-tailed test was conducted to determine whether our a priori hypothesis that the correlation between the beta-weight values in MD and the hippocampus would be anti-correlated in females (but not in males).

## 3. Results

There was no significant difference between the spatial memory accuracy of the female participants (74.25 ± 0.94%, mean ± SE) and the male participants (73.42 ± 1.22%, *t*(34) < 1).

**Figure 2:**
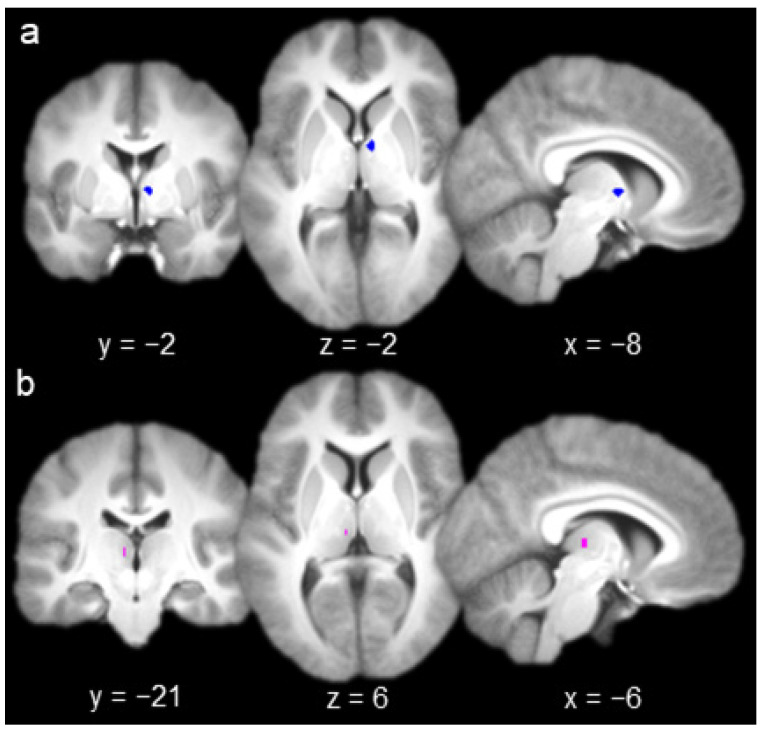
(**a**) Anterior thalamus seed (left, coronal slice; middle, axial slice; right, sagittal slice). (**b**) Mediodorsal thalamus seed.

The group contrast of hits versus misses produced one activation in AT (Figure 2a; x = 6, y = −2, z = 2) and one activation in MD (Figure 2b; x = −6, y = −21, z = 6). A 3 mm sphere centered at each of these coordinates (containing six voxels) was used as the seed for whole-brain functional connectivity analyses with each nucleus (Figure 2). All of the voxels comprising each seed were contained within their respective nuclei (according to Najdenovska et al.’s anatomic atlas [35,36]; see Methods, Section 2.3).

**Figure 3:**
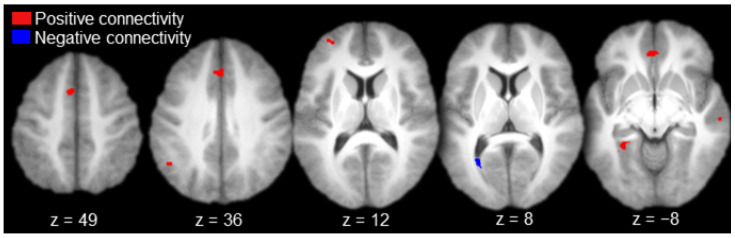
Anterior thalamus functional connectivity results (axial slices; key to the upper left).

Regions functionally connected to AT with positive magnitude (i.e., positive connectivity) included the left anterior prefrontal cortex, left medial prefrontal cortex, bilateral anterior cingulate gyrus, left inferior parietal cortex (angular gyrus), right lateral temporal cortex (superior temporal sulcus), and the left parahippocampal cortex (Table 1 and Figure 3, in red). There was a single activation of negative functional connectivity with AT in the left striate cortex (calcarine sulcus; Table 1 and Figure 3, in blue).

**Figure 4:**
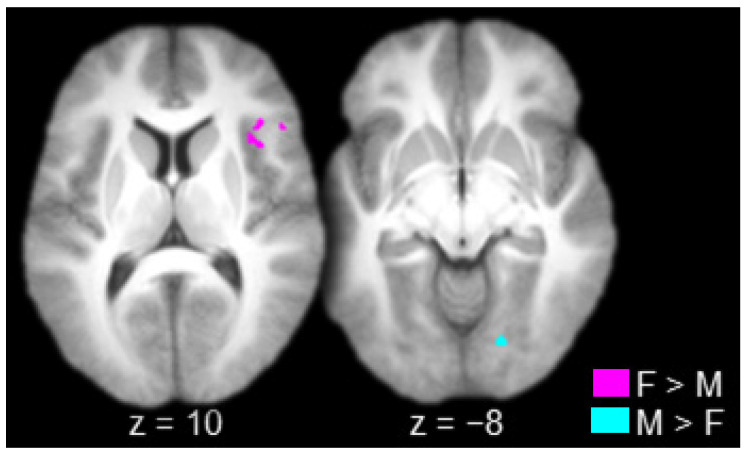
Regions connected to the anterior thalamus to a greater extent in females (F) than in males (M; in violet) and in males than in females (in cyan; axial slices; key at the bottom right).

The contrast between females and males produced one connected activation that spanned the right lateral prefrontal cortex (inferior frontal gyrus) and the right insula (Table 2 and Figure 4, in violet). The contrast between males and females produced one connected activation in the right striate/extrastriate cortex (calcarine sulcus/lingual gyrus; Table 2 and Figure 4, in cyan). 

**Figure 5:**
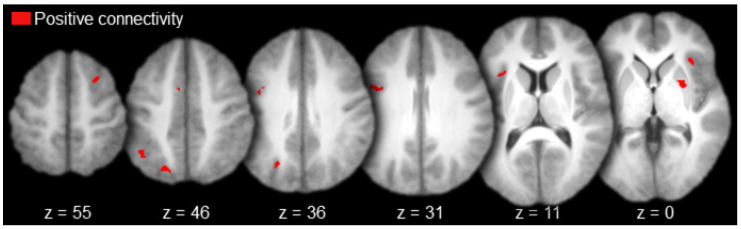
Mediodorsal thalamus functional connectivity results (axial slices; key to the upper left).

Regions with positive functional connectivity to MD included the bilateral lateral prefrontal cortex (right superior frontal sulcus and left precentral sulcus), left medial prefrontal cortex, left superior parietal cortex (intraparietal sulcus), bilateral insula, and the right putamen (Table 3 and Figure 5). There were no regions with negative functional connectivity to MD.

**Figure 6:**
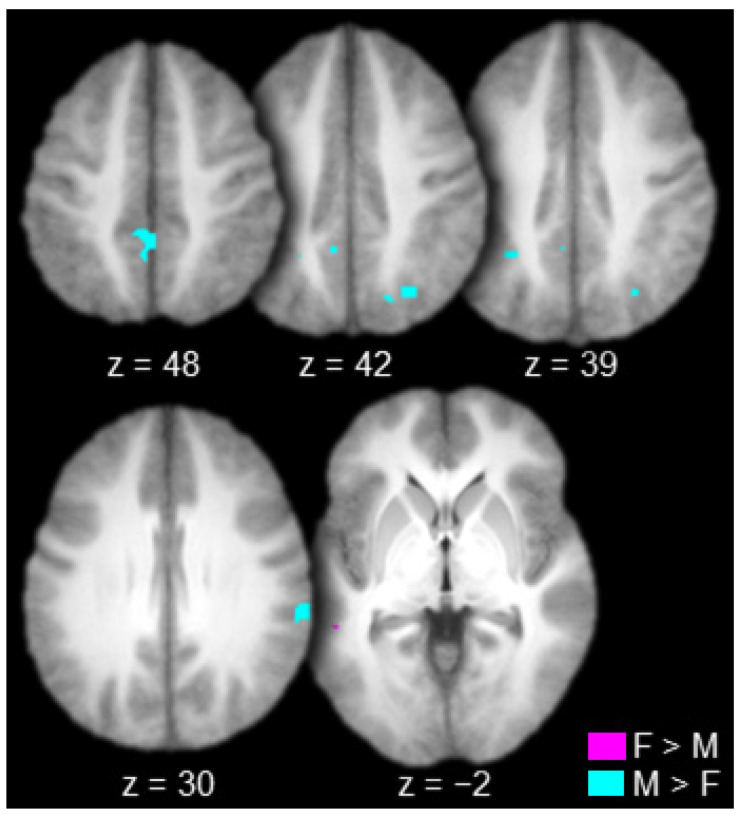
Regions functionally connected to the mediodorsal thalamus to a greater extent in females (F) than in males (M, in violet) and in males than in females (in cyan; axial slices; key at the bottom right).

The contrast between females and males produced one connected activation in the left lateral temporal cortex (left superior temporal sulcus; Table 4 and Figure 6, in violet). The contrast between males and females produced many connected activations including the right inferior parietal cortex (supramarginal gyrus), bilateral superior parietal cortex (bilateral intraparietal sulcus and bilateral precuneus), and the bilateral posterior cingulate gyrus (Table 4 and Figure 6, in cyan).

**Figure 7:**
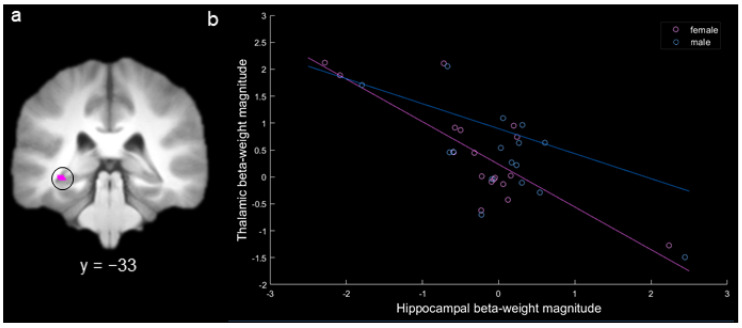
(**a**) Hippocampal activation produced by the contrast female hits > misses (circled). (**b**) Correlation between the hit – miss beta-weight values for the hippocampal region-of-interest (ROI) activation and mediodorsal thalamic activation for females (in pink) and for males (in blue; each point represents a participant; key at the upper right).

Based on our specific hypothesis regarding thalamic inhibition of the hippocampus in females, we conducted a targeted ROI analysis to determine whether there was a negative correlation between activity in the thalamus and hippocampus for females during spatial long-term memory. In the MD gPPI contrast of hits versus misses for females, there was a single activation with negative functional connectivity with the CA1 region of the left hippocampus (x = −34, y = −32, z = −6; there were no other significant connected activations for females or males). A 3 mm sphere was extracted around the peak of this activation to create a hippocampal ROI (Figure 7a). Activity in this ROI was significantly negatively correlated with activity in MD for females (*r* = −0.80, Bonferonni corrected *p* < 0.0005) but not for males (*r* = −0.25, Bonferonni corrected *p* > 0.20; Figure 7b), and the female correlation was significantly more negative than the male correlation (*p* < 0.01). The identical pattern of results was obtained using a Spearman correlation. To determine whether the significant negative correlation for females was driven by the three participants with more extreme beta-weight values (i.e., hippocampal beta-weight values below −2 or above 2), we conducted a follow-up correlation with these participants removed. With these three participants removed from the analysis, the negative correlation for females remained significant (*r* = −0.51, *p* < 0.05).

## 4. Discussion

In the present study, we found that AT had positive functional connectivity with the prefrontal cortex (anterior prefrontal cortex and medial prefrontal cortex), the anterior cingulate cortex, inferior parietal cortex (angular gyrus), super temporal cortex (superior temporal sulcus), parahippocampal cortex, and the inferotemporal cortex (fusiform gyrus), along with negative connectivity with V1 (i.e., striate cortex). Many of the positively connected activations are known to share anatomic connections with AT, including the prefrontal cortex [5] and regions of the Papez circuit, which is thought to support explicit memory [42]. Both the cingulate cortex and the parahippocampal cortex (two regions functionally connected to AT in the current study) are critical elements of the Papez circuit, as AT receives inputs from the hippocampus and mamillary bodies and relays these inputs to the cingulate gyrus, parahippocampal gyrus, and back to the hippocampus [43].

The parahippocampal cortex has been linked to many cognitive processes, including visuospatial processing and episodic memory and is particularly important for processing item context [44]. Parahippocampal cortex activation in the current study is most likely due to the nature of the spatial memory task, which requires an item (abstract shape) to be associated with a given context (spatial location). Although direct connections between AT and angular gyrus are not known to exist in humans, anatomical tracer studies in non-human primates have identified direct connections between the parahippocampal gyrus and the angular gyrus [45]. Moreover, connectivity between the parahippocampal cortex and the angular gyrus has been shown to increase during the identification of novel objects [46]. Thus, activation of the angular gyrus in the connectivity map may be due to downstream activation from the other components of AT circuit (such as the parahippocampal cortex).

In the direct comparison of females and males, females produced a greater magnitude of connected activity with AT in the lateral prefrontal cortex (inferior frontal gyrus) and insula. The lateral prefrontal cortex is often implicated in spatial long-term memory and has been thought to aid in selection of memory contents either by inhibiting related items or selecting relevant targets [32,47]. Males produced a greater magnitude of activity in the striate and extrastriate cortex (calcarine sulcus and lingual gyrus). Such activity in early visual processing regions can be assumed to reflect reactivation of early visual contents during memory retrieval [48]. Thus, males may rely on early visual processing during long-term memory construction than females. This is supported by recent evidence from a long-term memory meta-analysis that identified greater activity in visual regions for males compared to that in females across a variety of long-term memory types [27]. It has been hypothesized that AT is involved in the selection of memory contents [16]. Thus, AT may aid in selecting memory contents in females by inhibiting unrelated or closely related memory items, whereas AT may aid in selecting memory contents in males by selecting relevant visual memory components. The current findings suggest that this operation may involve coordination between AT and the lateral prefrontal cortex in females and between AT and the striate/extrastriate cortex in males.

MD had positive functional connectivity with the prefrontal cortex (superior frontal sulcus, precentral sulcus, and medial prefrontal cortex), superior parietal cortex (intraparietal sulcus), insula, and the putamen. MD shares dense reciprocal connections with the prefrontal cortex, thus, the current functional results are supported by a collection of anatomic evidence [10]. Although the intraparietal sulcus does not share any direct connections with MD, it is often activated in long-term memory processes, and this region may be related to sustained visual attention [49]. In the current task, the intraparietal sulcus likely mediates attention to the relevant aspects of a memory. MD has recently been shown to amplify prefrontal cortex connectivity in rodents, effectively sustaining attentional control [50]. Thus, MD may play a role in sustaining attention in humans as well via connections with the prefrontal cortex and intraparietal sulcus. This is a topic of future research. 

In the direct comparison of females and males, females produced a greater magnitude of connected activity with MD in the superior temporal cortex (superior temporal sulcus). The left superior temporal cortex has been associated with language processing [51]. It may be that during spatial long-term memory, females evoke a verbal retrieval strategy, which involves activity in the superior temporal cortex. This hypothesis is in line with literature that suggests that females utilize verbal memory strategies to a greater extent than males do [19,26,29]. Males produced a greater magnitude of activity in the inferior parietal cortex (supramarginal gyrus), superior parietal cortex (intraparietal sulcus and precuneus), and the posterior cingulate gyrus. A greater magnitude of activity in the intraparietal sulcus for males may suggest greater attention to memory contents compared to that in females. Overall, males produced a greater number of connected activations with MD relative to females. Thus, outside of specific hypotheses regarding the activation of particular brain regions, it may be that males produce overall greater activity during spatial long-term memory compared to females to achieve the same behavioral result. The “neural efficiency hypothesis,” is often cited in sex difference studies of long-term memory to potentially explain greater levels of neural activity in males compared to that in females (for a review, see [27]). Although many of the regions differentially connected for females and males do not share direct connections with MD, it is possible that these are regulated secondarily by other regions directly connected to this thalamic nucleus. 

Recent evidence in rodents has identified that inactivation of the thalamic–hippocampal pathway can rescue hippocampal activity and memory performance in female (but not in male) mice [30]. Based on this evidence, we hypothesized that activity in the thalamus would be anti-correlated with activity in the hippocampus for females, but not for males, which is what was observed. These results suggest that MD may play an inhibitory role in regulating hippocampal activation during spatial long-term memory that is specific to females. The hippocampal activation was localized to the CA1 subregion of the hippocampus [32,52], which has previously been associated with autobiographical memory (a cognitive process closely related to spatial memory). Since MD does not share any direct connections with the hippocampus, this inhibition may take place via a secondary structure, such as the prefrontal cortex [47]. Despite the lack of direct connections, functional connectivity between MD and subregions of the medial temporal lobe have previously been reported during long-term memory [14], including with the hippocampus [15]. The current results provide evidence that MD regulates hippocampal activation in a manner that is specific to females. The relationship between MD and hippocampal inactivation may explain why females often employ verbal strategies in tasks that are not necessarily verbal in nature [26]. Of course, as we cannot assess causality in the current study, it may also be the case that hippocampal inhibition is a consequence of the employment of verbal strategies in females. This hippocampal inhibition may also explain why the magnitude of hippocampal activity during long-term memory is sometimes greater in males than in females [24,26] and why females more often employ verbal strategies [19,29]. More generally, these results support the hypothesis that MD aids in the selection of a memory retrieval strategy [16]. 

The current study has some limitations. First, data were collected on two different MRI scanners, which would be expected to increase variance and yield null results. Of relevance, a recent study investigated scanner reliability during resting-state scans collected from three different scanners (Siemens Trio 3T, GE 3T HDx, and GE 3T discovery) that were analyzed using three different functional connectivity methods (seed based, intrinsic connectivity distribution, and matrix connectivity; [53]). There were no major variability effects in the results of the connectivity analyses based on the scanner location (i.e., site), scanner manufacturer, or time of day the scans were collected. Moreover, the effect of subject was found to be much greater than any of the other measured effects. It was noted that when using a single 5 min scan as a sample, the reliability in connectivity measures was poor; however, when the duration of the scan was increased to 25 min, the reliability increased. It was suggested that when collecting data from multiple scanners, a minimum scan time of 25 min should be employed, and that this is sufficient to aggregate functional connectivity data across multiple scanners. As the average scan time in the current study was 42.25 min, the results of the current study are unlikely to be affected by differences across the two scanners. Noble et al. [53] also found that seed-based connectivity approaches, which the current analysis employed, are more reliable than other types of connectivity approaches. It should also be noted that between-scanner variability would be expected to increase variance and produce null results. Since significant (rather than null) results were observed in the present study, such variability was not a major issue. That said, acquiring data on two different scanners was not ideal, and this is a limitation of the current study. Second, the correlation analysis contained 18 participants per group. It would of course have been preferable for such a correlation analyses to have a larger *N* [54,55]. It is not uncommon, however, for correlation analyses in long-term memory fMRI studies to have sample sizes comparable to that of the present study [56,57,58,59,60,61]. Nevertheless, the correlation results in the current study should be viewed as preliminary, and future studies should employ larger sample sizes.

## 5. Conclusions

The current results contribute to a growing literature supporting the role of the thalamus in cognition. Functional connectivity was identified between AT and MD and many regions associated with memory including the prefrontal cortex, parietal cortex, visual processing regions, hippocampus, and the parahippocampal cortex. Many of the regions functionally connected to AT and MD shared direct connections supported by anatomic evidence, while others may be related to these nuclei via secondary connections. Moreover, we identified that sex differences exist in functional connectivity between MD and the hippocampus, with greater magnitudes of activity in MD relating to lower magnitudes of activity in the hippocampus for females (but not for males). More broadly, the present results point to an important role of the thalamus in human memory, which to some extent is modulated by sex. These sex differences argue against the common practice of collapsing across sex in cognitive neuroscience studies.

## Figures and Tables

**Table 1:** Regions functionally connected to the anterior thalamus during spatial memory hits > misses.

Region	BA	x	y	z	k
*All participants*					
*Positive activations*					
L. Anterior Prefrontal Cortex	10	−32	51	12	31
L. Medial Prefrontal Cortex	6	−5	11	48	50
Bilateral Anterior Cingulate Gyrus	32	0	24	38	30
Bilateral Anterior Cingulate Gyrus	32	0	39	−10	32
L. Angular Gyrus	39	−44	−54	34	51
R. Superior Temporal Sulcus	21/22	60	−21	−7	29
L. Parahippocampal Cortex	19/37	−27	−46	−-7	27
*Negative activations*					
L. Calcarine Sulcus	17	−26	−62	8	30

BA refers to Brodmann area and Montreal Neurological Institute (MNI). Coordinate (x, y, z) refers to the center of each activation. L., left; R., right.

**Table 2:** Regions differentially connected to the anterior thalamus between females and males during spatial memory hits > misses.

Region	BA	x	y	z	*k*
*Female (Hits > Misses) > Male (Hits > Misses)*					
*Positive activations*					
R. Inferior Frontal Gyrus/Insula	44/45	39	25	7	74
*Male (Hits > Misses) > Female (Hits > Misses)* *Negative activations*					
L. Calcarine Sulcus/Lingual Gyrus	17/18	19	−76	−5	32

BA refers to Brodmann area and Montreal Neurological Institute (MNI). coordinate (x, y, z) refers to the center of each activation. L., left; R., right.

**Table 3:** Regions functionally connected to the mediodorsal thalamus during spatial memory hits > misses.

Region	BA	x	y	z	*k*
*All participants*					
*Positive activations*					
R. Superior Frontal Sulcus	6/8	27	12	53	29
L. Precentral Sulcus	6	−37	3	32	48
L. Medial Prefrontal Cortex	6	−7	8	50	37
L. Intraparietal Sulcus	19/39	−19	−68	45	50
L. Intraparietal Sulcus	7/40	−39	−55	45	27
L. Insula	−	−31	16	4	32
R. Insula	−	34	24	−2	43
R. Putamen	−	27	3	0	41
*Negative activations*					
No activations					

BA refers to Brodmann area and Montreal Neurological Institute (MNI). coordinate (x, y, z) refers to the center of each activation. L., left; R., right.

**Table 4:** Regions differentially connected to the mediodorsal thalamus between females and males during spatial memory hits > misses.

Region	BA	x	y	z	*k*
*Female (Hits > Misses) > Male (Hits > Misses)*					
*Positive activations*					
L. Superior Temporal Sulcus	22	−48	−41	−2	31
*Male (Hits > Misses) > Female (Hits > Misses)* *Negative activations*					
R. Supramarginal Gyrus	40	61	−31	29	40
L. Intraparietal Sulcus	7/40	−28	−48	40	24
R. Intraparietal Sulcus	19/39	28	−67	42	37
Bilateral Precuneus/Post. Cingulate Gyrus	7/31	0	−42	48	55

BA refers to Brodmann area and Montreal Neurological Institute (MNI) coordinate. (x, y, z) refers to the center of each activation. L., left; R., right; Post., posterior.
